# Feasibility of the development and psychometric properties of a standardized screening instrument for mental disorders in patients with suspected rare diseases: results of the ZSE-DUO study

**DOI:** 10.3389/fpsyt.2025.1624474

**Published:** 2025-11-10

**Authors:** Lilly Sophia Brandstetter, Kathrin Ungethüm, Peter U. Heuschmann, Lisa Pfister, Jürgen Deckert, Martina de Zwaan, Oliver Tüscher, Stefanie Witt, Christian Krauth, Alexandra Berger, Mark Berneburg, Anna Deibele, Jan Dieris-Hirche, Gereon Heuft, Christiane Imhof, Jörg Bernhard Schulz, Stephan Zipfel, Helge Hebestreit, Kirsten Haas

**Affiliations:** 1 Institute for Clinical Epidemiology and Biometry, Julius-Maximilian University Würzburg, Würzburg, Germany; 2 Institute of Medical Data Science, University Hospital Würzburg, Würzburg, Germany; 3 Clinical Trial Centre Würzburg, University Hospital Würzburg, Würzburg, Germany; 4 Centre for Rare Diseases, University Hospital Würzburg, Würzburg, Germany; 5 Department of Psychiatry, Psychosomatics and Psychotherapy, University Hospital Würzburg, Würzburg, Germany; 6 Department of Psychosomatic Medicine and Psychotherapy, Hannover Medical School, Hannover, Germany; 7 Department of Psychiatry and Psychotherapy, University Medical Center Mainz, Mainz, Germany; 8 Center for Psychosocial Medicine, Department of Medical Psychology, University Medical Center Hamburg-Eppendorf, Hamburg, Germany; 9 Centre for Health Economics Research Hannover and Institute for Epidemiology, Social Medicine and Health Systems Research, Hannover Medical School, Hannover, Germany; 10 Frankfurt Reference Centre for Rare Diseases, University Hospital Frankfurt, Goethe-University Frankfurt, Frankfurt, Germany; 11 Centre for Rare Diseases Regensburg, University Hospital Regensburg, Regensburg, Germany; 12 Department of Psychosomatic Medicine and Psychotherapy, University Hospital Magdeburg, Magdeburg, Germany; 13 Department of Psychosomatic Medicine and Psychotherapy, LWL-University Hospital, Ruhr University Bochum, Bochum, Germany; 14 Department of Psychosomatic Medicine Und Psychotherapy, University Hospital Münster, Münster, Germany; 15 Department of Psychosomatic Medicine and Psychotherapy, Ulm University Medical Center, Ulm, Germany; 16 Department of Neurology, RWTH Aachen University, Aachen, Germany; 17 Center for Rare Diseases, Medical Faculty, RWTH Aachen University, Aachen, Germany; 18 Department of Psychosomatic Medicine and Psychotherapy, University of Tübingen, Tübingen, Germany; 19 Department of Pediatrics, University Hospital Würzburg, Würzburg, Germany

**Keywords:** rare diseases, mental health, questionnaire development, screening instrument, confirmatory factor analysis

## Abstract

**Introduction:**

Patients presenting in centers for rare diseases (CRDs) show complex symptoms, requiring multiprofessional expertise. Many patients suffer from unnoticed mental disorders. Currently, mental health experts (MHEs) are rarely involved in the diagnostic process. The aim of this study was to test the feasibility of developing a new short screening instrument for mental disorders and to test its predictive value.

**Methods:**

Data were derived from 1,300 adult patients participating in ZSE-DUO (dual guidance structure in centers for rare diseases), a multicenter study in 11 CRDs (funded by G-BA, Grant 01NVF17031), evaluating the benefit of involving an MHE in the diagnostic process. Patients completed standardized questionnaires on anxiety [the 7-item Generalized Anxiety Disorder (GAD-7)], depression [the 9-item Patient Health Questionnaire (PHQ-9)], dissociation [4-item Dissociation-Tension Scale (DSS-4)], psychopathology [Symptom-Checklist K-9 (SCL-K-9)], and quality of life [12-item Short Form Health Survey (SF-12) and EQ-5D-5L] prior to and during their first CRD visit as part of the routine assessment necessary for a first contact at a CRD. Exploratory factor analysis (EFA) was performed for item reduction. The reliability of the factor structure was assessed using Cronbach’s α. Model fit was tested using confirmatory factor analysis (CFA). The predictive value of the new screening instrument was tested by calculating a receiver operating characteristic (ROC) curve using the diagnoses from the MHE.

**Results:**

The extracted 18-item model had a four-factor structure with acceptable to high reliability. The extracted mental health dimensions were 1) anxiety and depression, 2) mobility and activities of daily living, 3) energy or fatigue, and 4) dissociation. Excluding the dimension “dissociation” (well assessed using the DSS-4 separately), a summary score was calculated ranging from 0 to 45, with the lowest score representing the best mental health. In the CFA, the model fit indices for the extracted factor structure did meet the established thresholds. The predictive value of the new screening instrument on any mental disorder was moderate [area under the curve (AUC)=0.68; 95% CI=0.64–0.73].

**Conclusion:**

The design of a new short screening instrument for mental disorders in patients presenting at CRDs was feasible. Furthermore, the new short screening instrument may help MHEs to identify patients in need of a more thorough screening and adapted care, particularly in identifying patients with potential depressive disorders. However, due to the heterogeneity of the mental disorders in this patient group, in addition, individual assessment of patients by MHEs is necessary.

## 1 Introduction

For patients with persistent symptoms of unclear origin, centers for rare diseases (CRDs) provide an extensive diagnostic process, including expert clinical evaluation, in-depth phenotyping, and whole exome or genome sequencing with innovative analyses (1). The symptoms of unclear origin—diagnoses can include rare diseases (RDs) (RDs=diseases that affect no more than one in every 2,000 people (2)) and non-RD—are often very complex. As a result, the diagnosis is often delayed, i.e., several years after the first symptoms appear (3). Therefore, patients may experience frustration and self-doubt (4).

In addition to complex somatic symptoms, patients presenting to CRDs often suffer from unrecognized and untreated psychopathological symptoms or mental disorders (2, 5). The ZSE-DUO study (*Dual Guidance Structure for Evaluation of Patients with Unclear Diagnosis in Centers for Rare Diseases*) found that approximately 30% of the patients presenting to CRDs suffered from a mental disorder (6). The psychopathological symptoms can be part of the clinical presentation of some rare (RDs) and non-rare diseases (non-RDs) (7). They may develop in the often long and tedious course of searching for a diagnosis, can independently co-occur with a (non) RD, or may even mimic the signs of an RD (8).

The extensive diagnostic process at CRDs often requires the collaboration of several experts from different clinical fields. However, to this day, mental health experts (MHEs) are rarely involved in the diagnostic process. Within the multicenter ZSE-DUO study in 11 CRDs, the benefit of permanently involving an MHE in the diagnostic process at CRDs was evaluated, suggesting that including an MHE in the entire diagnostic process should become an integral part of the assessment of patients with symptoms of unclear origin (9). In order to target care to patients with a high psychopathological burden, a screening instrument to direct MHE resources to these patients may be useful in clinical routine. To the best of our knowledge, there is an absence of a short screening tool available to identify patients with suspected mental disorders. This is especially evident in the identification of such patients prior to an in-depth consultation with an MHE.

To date, structured diagnostic instruments for mental disorders such as the Structured Clinical Interview for DSM-5 (SCID-5-CV) exist (10). Nevertheless, these tools are quite time-consuming (1–2 hours) and can only be applied by MHEs. Alternatively, instruments to screen for psychological distress, like the Symptom-Checklist K-9 (SCL-K-9), can be applied (11), which, however, does not allow a differential diagnosis. In order to screen for specific mental disorders, it is necessary to employ other standardized instruments, such as the 9-item version of the Patient Health Questionnaire (PHQ-9) (12, 13) for depressive symptoms, or the 7-item version of the Generalized Anxiety Disorder (GAD-7) questionnaire (14) for anxiety symptoms. To screen for multiple mental disorders simultaneously, a multitude of instruments would need to be presented to patients, which could increase their burden.

Therefore, as part of the ZSE-DUO study, the aim of this study was to test the feasibility of developing a new short screening instrument for mental disorders, based on established screening instruments, and to test its psychometric properties and predictive value.

## 2 Methods

### 2.1 Ethics approval and consent to participate

The study was performed in full accordance with the principles of the “Declaration of Helsinki” (as amended in Tokyo, Venice, and Hong Kong) and with the EC/ICH Guidelines on Good Clinical Practice. All methods of the study were fully approved by the ethics committee of the Medical Faculty of the University of Würzburg (reference number 132–18). The main ethical approval was confirmed by the ethics committees of all cooperating centers. Written informed consent was obtained from all participants or their legal representative. The responsible data protection officer accepted the data management concept.

### 2.2 Participants, study design, data collection process, and database

Data were derived from adults participating in the ZSE-DUO study (ClinicalTrials.gov identifier: NCT03563677). ZSE-DUO is a multicenter study in 11 CRDs, funded by the Federal Joint Committee (G-BA) under grant number 01NVF17031, evaluating the benefit of permanently involving an MHE in the diagnostic process at CRDs. ZSE-DUO is a prospective, controlled trial with a two-phase cohort design—the first cohort was enrolled in the control group (CG) and a subsequent second cohort in the intervention group (IG)—with the former receiving standard care and the latter additionally undergoing consultation with an MHE. In consequence of the characteristics of the patients, the settings, and the interventions, a sequential cohort design with the IG following the CG was deemed to be the optimal approach in comparison with a randomized trial. This decision was made due to the inability to blind participants and team members and the potential for cross-contamination between groups in a randomized design (9). Details on inclusion/exclusion criteria, recruitment procedures, and the standard and innovative care provided to the CG and IG are published elsewhere (9). Furthermore, details on the characteristics of the study population were described by Hebestreit et al. (2023) (6).

**Figure 1:**
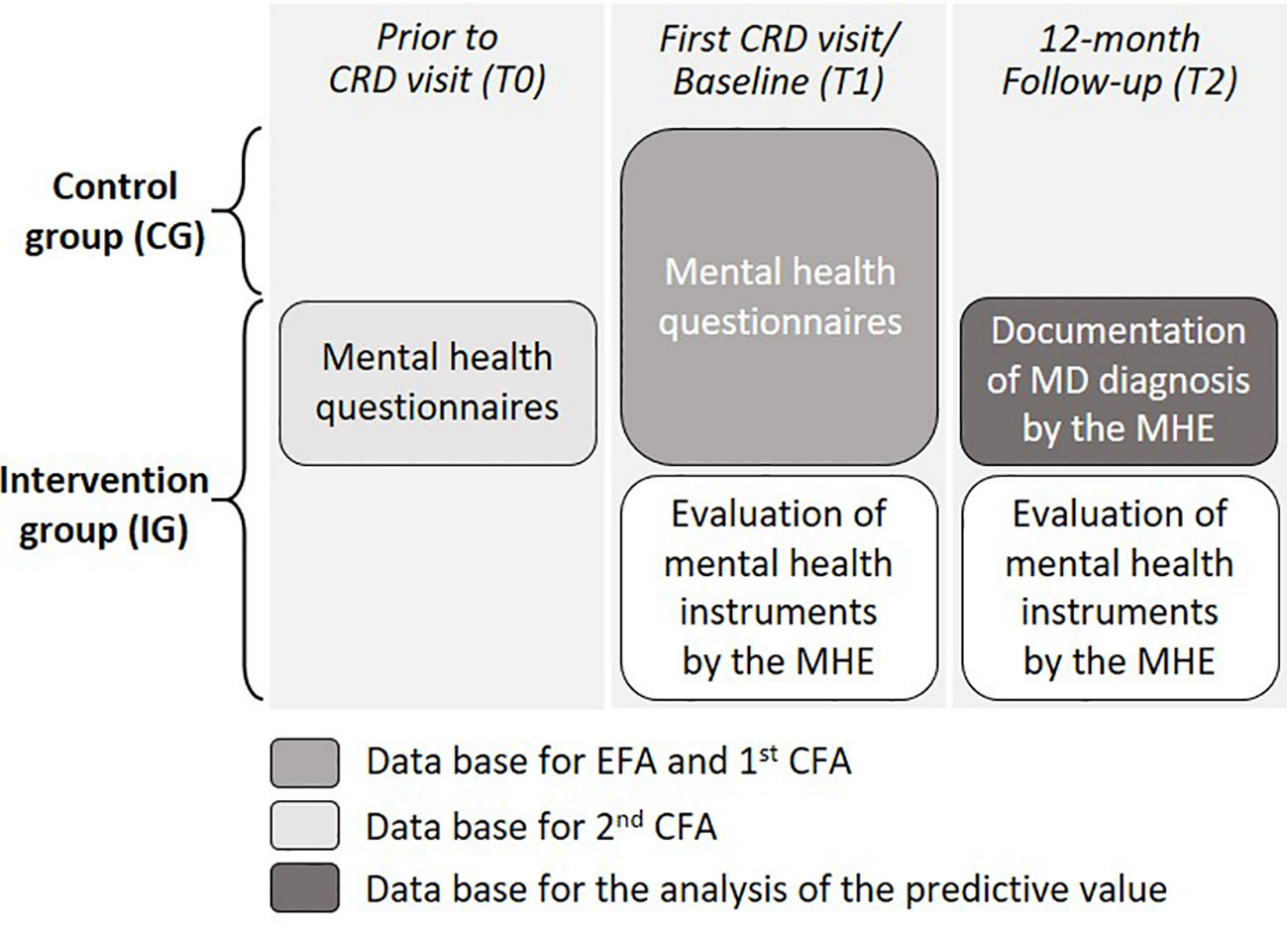
Data collection and overview of database for analyses.

For all patients, it was necessary that all medical records and a physician referral were provided to the CRDs. After evaluation of these records at the CRDs, patients were invited to their first visit. In the frame of this invitation, patients send the standardized questionnaires on anxiety (10), depression (11), dissociative symptoms (12), general psychopathological symptoms (13), and health-related quality of life (14, 15) (detailed descriptions below) prior to the first CRD visit (T0) (**Figure 1**) as part of the routine assessment necessary for a first contact at a CRD. Thereafter, the patients returned the completed questionnaires to the CRD for review by the MHE.

In both the CG and IG, patients completed the same standardized questionnaires on the day of their first visit at a CRD (T1) (**Figure 1**). Patients in both groups were followed up for 12 months. Within these 12 months after patients’ first personal consultation at a CRD (T2), the MHE documented the patients’ diagnoses of mental disorders (9) (**Figure 1**). To reflect the idea that the new short screening instrument should assist the MHE in identifying patients in need, both confirmed and suspected mental disorders were included in the analyses. Furthermore, patients with suspected diagnoses were referred to routine care for further diagnosis. Hence, not all diagnoses may have been confirmed during the 12-month follow-up, and some diagnoses may not have been communicated from routine care institutions to the CRDs.

To support the item selection for the new short screening instrument, in the IG, the MHE completed a questionnaire evaluating the questionnaires on mental health at baseline (T1) and 12 months after patients’ first personal consultation at a CRD (T2) (**Figure 1**; for detailed description, see Section 2.4).

### 2.3 Standardized instruments on mental health

Standardized instruments on mental health were chosen based on expert opinion within the ZSE-DUO study.

#### 2.3.1 Health-related quality of life

##### 2.3.1.1 EQ-5D-5L

The EQ-5D-5L is a generic measurement tool developed by the EuroQol Group (1990) for the assessment of quality of life (QoL) in the five dimensions—mobility, self-care, usual activities, pain, and anxiety—on the day of the survey (15). Each dimension is measured by one item in five levels ranging from 1=“no problems” to 5=“extreme problems”.

##### 2.3.1.2 EQ-Visual Analogue Scale

The EQ Visual Analogue Scale (EQ-VAS) represents a quantitative patient-reported measure of perceived health on a visual analogue scale (0–100) with endpoints labeled “the worst you can imagine” and “the best health you can imagine” (15).

##### 2.3.1.3 12-Item Short Form Health Survey

The 12-item Short Form Health Survey (SF-12) assesses physical and mental health during the last 2 weeks prior to the survey (16). The 12 items comprising general health perception, physical functioning, role-physical, role-mental, bodily pain, mental health, vitality, and social functioning are measured on 3- to 6-point Likert scales or as yes/no questions.

#### 2.3.2 Anxiety—Generalized Anxiety Disorder 7

The GAD-7 is a 7-item questionnaire for the assessment of general anxiety disorder (GAD) symptoms in the last 2 weeks prior to the survey (14). Each item is measured on a 4-point Likert scale ranging from 0=“not at all” to 3=“nearly every day”.

#### 2.3.3 Depression—Patient Health Questionnaire 9

The PHQ-9 assesses depressive symptoms in the last 2 weeks prior to the survey (12, 13). Each item is measured on a 4-point Likert scale ranging from 0=“not at all” to 3=“nearly every day”.

#### 2.3.4 General psychopathological symptoms—Symptom-Checklist K-9

The German SCL-K-9 (17) is a short version of the SCL-90-Revised (11), a multidisciplinary instrument to screen for psychological distress. The SCL-K-9 comprises nine items asking about complaints in the last 7 days prior to the survey. Each item is measured on a 5-point Likert scale ranging from 0=“not at all” to 4=“extremely”.

#### 2.3.5 Dissociative symptoms—Dissociation-Tension Scale

The 4-item short version of the Dissociation-Tension Scale (DSS-4) is based on the DSS-acute (18) and assesses dissociative symptoms in the last 7 days prior to the survey (19). Each item is measured on an 11-point Likert scale ranging from 0=“no impression” to 10=“strong impression”.

#### 2.3.6 Life satisfaction

Life satisfaction was assessed using a single item on an 11-point Likert scale ranging from 0=“not satisfied at all” to 10=“completely satisfied” (20).

### 2.4 Evaluation of mental health instruments by the MHE

To evaluate the standardized questionnaires on mental health, a self-developed questionnaire was filled in by the MHEs. In this questionnaire, the MHEs were asked whether they had considered patients’ first questionnaire on mental health [completed prior to the first visit at a CRD (T0, see **Figure 1**)] in their assessment (yes/no), and which of the included instruments they considered helpful or directive for their conclusions (yes/no for each instrument). They were further asked whether they had considered patients’ second questionnaire on mental health [completed during the first personal consultation at a CRD (T1, see **Figure 1**)] for their review (yes/no). In addition, the MHE were asked whether the implementation of these questionnaires was useful in general and, if so, at which time point in the diagnostic process (T0 or T1, or both; see **Figure 1**).

### 2.5 Statistical analyses

#### 2.5.1 Evaluation of mental health instruments by the MHE

The results of the evaluation of the mental health instruments by the MHE at baseline and 12 months after patients’ first personal consultation at a CRD are displayed as percentages.

#### 2.5.2 Item selection from standardized mental health instruments—exploratory factor analysis

Exploratory factor analyses (EFAs) were conducted to select items from standardized mental health instruments to be included in a new short screening instrument for patients presenting to CRDs.

The items life satisfaction, EQ-VAS, physical functioning, mental health, and social functioning from the SF-12 were inverted; thus, for every item, a lower score would represent a better mental health. Dichotomous items were excluded. All items from the mental health assessment in both groups at T1 were included in the EFA (for the database, see **Figure 1**). Data from both the IG and CG were included in the EFA and first confirmatory factor analysis (CFA; see Section 2.5.3) for several reasons: to address potential group differences due to the sequential study design, to achieve a sufficiently large sample size, and to ensure that the new screening instrument was tested at a different time point than the one used for goodness of fit. The factor analyses were conducted following best practices described by Costello and Osborne (21) and Cabrera-Nguyen (22).

The suitability of the data for the EFA was examined based on the Kaiser–Meyer–Olkin (KMO) statistics of sampling adequacy and Bartlett’s test of sphericity (23, 24). The factor extraction was performed using a varimax rotation with Kaiser normalization. Kaiser criterion and scree plot analysis were used to determine the number of factors to extract (25, 26). Although it could be assumed that the items and factors are strongly correlated, we decided to use varimax rotation (orthogonal rotation). This is because, given the expected correlations, we would not have been able to extract clear, simple structures. However, this was absolutely necessary in order to reduce the number of items.

We considered items with cross-loading values of ≥0.32 on at least two factors as problematic (21). These items were removed from the item set. Items with factor loadings of ≥0.60 were considered strong (27). Moreover, if two or more items covered the same mental health domain, as different standardized questionnaires with overlapping domains were used, only one item was kept for further analysis. The selection of items for the final set was based on factor loadings and on the responses of the MHE in the follow-up questionnaire (the item of the higher-ranked instrument was kept). For each of the extracted factors (respective mental health domains), the internal reliability was tested and reported as Cronbach’s α coefficients. The discriminant validity of the extracted factors was examined by evaluating the factor correlations (≥0.10=weak correlation, ≥0.30=moderate correlation, and ≥0.50=strong correlation). Based on the final item set, a short questionnaire was designed, and a summary score was calculated.

#### 2.5.3 Goodness of fit—confirmatory factor analysis

A CFA was conducted to assess the goodness of fit, internal validity, and consistency of the factor structure (respective mental health domains) extracted from the EFA. As this was a feasibility study to gain first insights into the possibility of developing a screening instrument for mental health problems in patients presenting at CRDs, the external validity was not tested. The factor analyses were conducted following best practices described by Brown (28), Costello and Osborne (21), and Cabrera-Nguyen (22). In the first CFA, the same database (T1) as for the EFA was used to test the extracted model (database see **Figure 1**). The model included all items from the final set and the respective factor structure resulting from the EFA. The goodness of fit was evaluated using the following indices: 1) Pearson’s χ^2^ test, reflecting model fit; 2) comparative fit index (CFI; ≥0.95/0.90 is good/acceptable) and Tucker–Lewis index (TLI; >0.95 is acceptable), reflecting incremental fit; 3) the root mean square error of approximation (RMSEA; ≤0.06/0.08 is good/acceptable), reflecting absolute fit; and 4) standardized root mean square residual (SRMR; ≤0.06/0.08 is good/acceptable). Applied threshold values are recommended by Hu and Bentler (29, 30) and validated by Brown (28) and Cabrera-Nguyen (22). Maximum likelihood (ML) estimation was applied to estimate the factor loadings, and the latent factors were standardized to allow free estimation of all factor loadings. In the second CFA, data from the IG at the time T0 were used, as T0 represents the point in time at which the new screening instrument is ultimately to be implemented. The model included all items from the final set and the respective factor structure resulting from the EFA.

#### 2.5.4 Discriminatory power of the new short screening instrument on mental health

Confirmed and suspected diagnoses of mental disorders in the IG were analyzed descriptively and presented as total numbers and percentages. The discriminatory power of the newly generated short screening instrument on mental health problems to predict the (confirmed/suspected) diagnosis of a mental disorder was tested using receiver operating characteristic (ROC) analyses (database see **Figure 1**). The results are presented as ROC curves (95% CI), and the area under the curve (AUC) is reported. All diagnoses of mental disorders documented for the IG (n=664) in the follow-up by the MHE (at T2) were taken into account. Mental disorders were defined using the ICD-10 code level 1, the F group. Taking into account potential class imbalance for the diagnoses of mental disorder, a precision-recall curve (PRC) and corresponding area under the curve (PR-AUC) were calculated. To compare the discriminatory power of the new screening instrument with that of the standardized instruments on mental health, ROC analyses were additionally performed for each standardized instrument. Additionally, to explore the discriminatory power of the new screening instrument for specific mental disorders, ROC analyses were performed for ICD-10 code level 1 F groups: 1) F30 and F33–F39, 2) F40–F41, and 3) F43.2 and F45.

To generate a cut-off score for the new screening instrument, Youden’s index was calculated as another key summary statistic of the ROC curve (31). Youden’s index (*J*) is defined as *J*=max*_c_* [Se (*c*) + Sp (*c*) − 1], with sensitivity Se, sphericity Sp, and cut-off point c. The cut-point that achieves this maximum is referred to as the optimal cut-point (c*) because it is the cut-point that optimizes the discriminatory power of the score when sensitivity and specificity are given equal weight. The extracted cut-off was then tested using the χ^2^ test.

All analyses were carried out using SPSS 29 for Windows (SPSS Inc., Chicago, IL, USA). The CFA was performed using the AMOS Graphics 28.0 program (32). PRC and PR-AUC were calculated in R 4.3.1 (33) using the R-package PRROC 1.3.1 (34).

## 3 Results

Data were derived from 1,300 adult patients (CG=636 and IG=664) participating in ZSE-DUO. The median age was 48 years (IQR=34–57), and 60.9% were female. The detailed description of the study population is published elsewhere (6).

### 3.1 Evaluation of mental health instruments by the MHE

**Figure 2:**
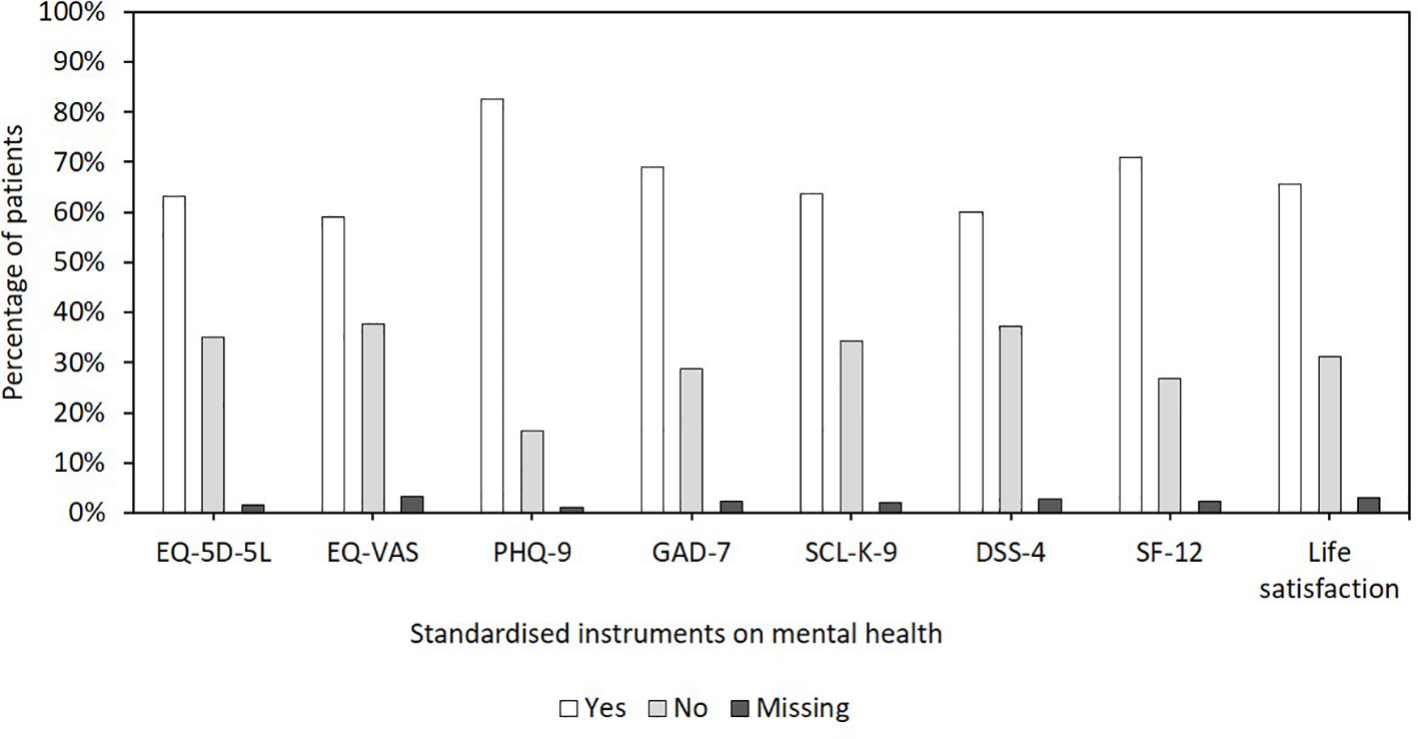
Responses of the mental health experts (MHEs) to the question of whether the respective instruments on mental health were helpful or directive in their assessment.

During the first visit in the IG (T1), the MHE took into account the standardized questionnaires on mental health (completed prior to patients’ CRD visit) of 87.8% of patients. **Figure 2** shows the responses of the MHEs to the question of whether the respective instruments were helpful or directive in their assessment. The PHQ-9 (72.6%), the SF-12 (62.5%), and the GAD-7 (60.5%) were the MHEs’ preferred questionnaires in most patients. In addition, for 56.6% of patients, the MHE also considered the mental health questionnaires completed by the patients at their first CRD visit as part of their medical history. In general, the MHE indicated that the use of mental health questionnaires was useful for 88.1% of the patients. In terms of time point, for most patients, the MHE preferred to use the questionnaires completed by the patients prior to their first visit at a CRD (T0) (78.8%) rather than the questionnaires completed by the patients during their CRD appointment (T1) (8.7%).

At follow-up (T2), the MHE stated that only for 49.8% of the patients were the mental health questionnaires directed at the process of symptom clarification. However, they reported that for 89.6% of patients, the use of these questionnaires is appropriate if implemented prior to patients’ first CRD visit (T0). For 78.0% and 7.5% of the patients, the MHE reported that the use of these questionnaires would be appropriate at the first visit (T1) or at additional time points in the course of the treatment, respectively.

### 3.2 Item selection from mental health instruments

**Figure 3:**
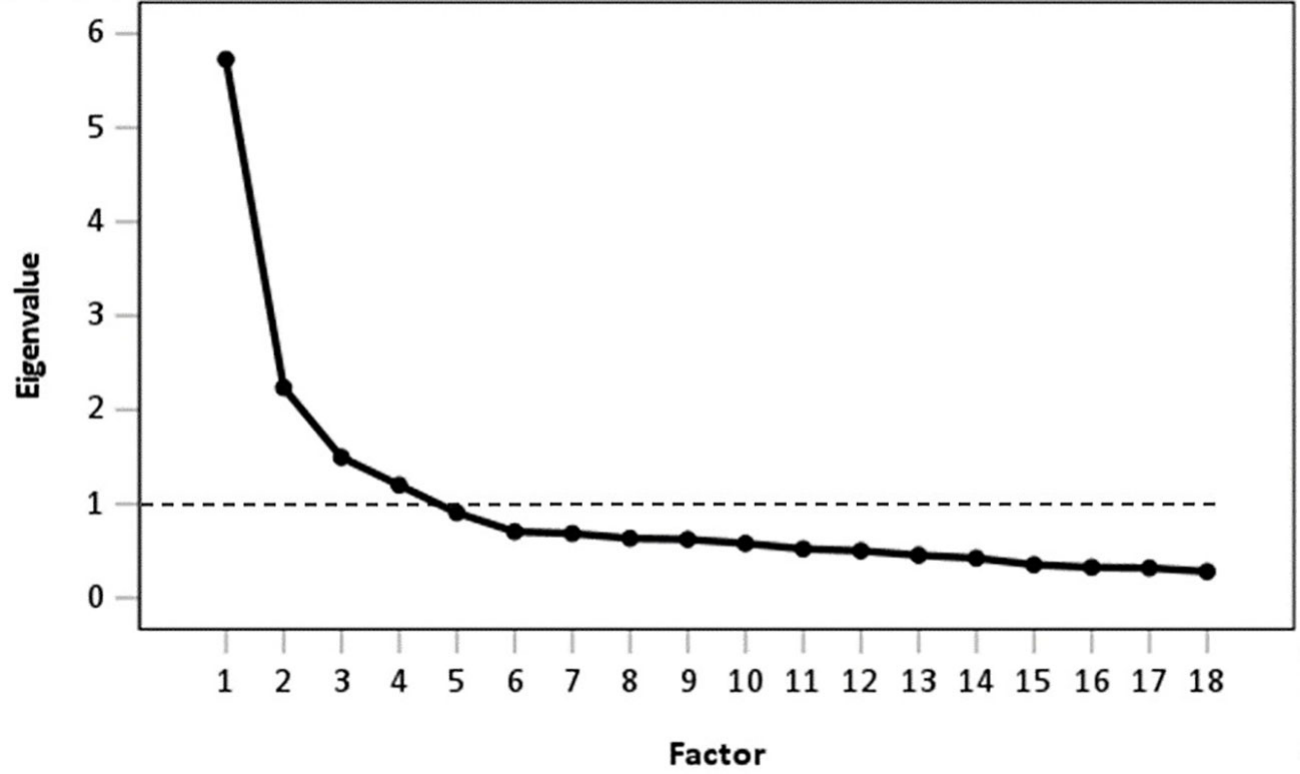
Scree plot and Kaiser criterion are shown by the black dashed line.

In the first EFA, all 44 items from the mental health instruments were included (**Supplementary Table 1**). The KMO measure of sampling adequacy was 0.96, and Bartlett’s test of sphericity was significant [χ^2^(946)=29,865.92; *p*<0.001], indicating adequate factorability. The Kaiser criterion method showed that the data contained six factors with eigenvalues greater than 1. The scree plot also suggested six factors. The following item reduction was based on the factor loadings in the rotated component matrix. Sixteen items with cross-loading values ≥0.32 were removed. The remaining 28 items were included in a second EFA (**Supplementary Table 2**). Both the Kaiser criterion method and the scree plot showed that the data contained four factors with eigenvalues greater than 1. After further reduction of three items due to cross-loadings ≥0.32, a third EFA (25 items) was conducted (**Supplementary Table 3**). Again, both approaches to determine the number of factors to extract supported a four-factor structure. A further seven items were excluded because they covered the same mental health domain as other items. Hence, the final item set consisted of 18 items. **Figure 3** shows the scree plot supporting the four-factor structure. These four factors accounted for 59.25% of the variance of all variables. The four extracted factors represent the mental health domains: 1) “anxiety/depression”, 2) “mobility/ability of daily life”, 3) “energy/fatigue”, and 4) “dissociation”. The final item set with the corresponding factor loadings and the factor structure is displayed in **Table 1**.

**Table 1:** Extracted factor structure with factor loadings, rotated to the varimax criterion.

Originating scale	Item	Factor/mental health domain			
		Anxiety/depression	Mobility/activities of daily life	Energy/fatigue	Dissociative symptoms
GAD-7	Feeling nervous, anxious, or on edge	0.72	0.12	0.20	0.09
	Not being able to stop or control worrying	0.81	0.11	0.17	0.07
	Becoming easily annoyed or irritable	0.61	0.05	0.23	0.06
	Feeling afraid, as if something awful may happen	0.78	0.02	0.06	0.18
PHQ-9	Feeling down, depressed, or hopeless	0.76	0.07	0.29	0.10
	Thoughts that you would be better off dead or of hurting yourself	0.62	0.11	−0.01	0.19
SCL-K-9	Emotional vulnerability	0.70	0.10	0.18	0.09
SF-12	Limited in climbing several flights of stairs	0.06	0.76	0.27	0.09
	Limited in moderate activities	0.01	0.80	0.23	0.07
EQ-5D-5L	Mobility	0.13	0.85	0.07	0.06
	Self-care	0.20	0.74	−0.07	0.09
PHQ-9	Trouble falling or staying asleep, or sleeping too much	0.25	0.08	0.66	0.07
	Feeling tired or having little energy	0.24	0.14	0.83	0.11
SF-12	Having a lot of energy	0.24	0.23	0.74	0.10
DSS-4	Sensation that body does not belong to you	0.29	0.18	0.16	0.59
	Problems with hearing/sounds coming from far away	0.10	0.06	0.10	0.75
	Sensation that people/things/world are not real	0.22	−0.02	0.03	0.71
	Sensation that body/body parts are insensitive to pain	−0.01	0.09	0.02	0.61

GAD-7, 7-item Generalized Anxiety Disorder; PHQ-9, 9-item Patient Health Questionnaire; SCL-K-9, Symptom-Checklist K-9; SF-12, 12-Item Short Form Health Survey; DSS-4, 4-item Dissociation-Tension Scale.

By adding up the resulting scores, a summary score for the newly generated short screening instrument was calculated. **Table 2** shows an overview of the items and scoring in the new screening instrument. The score ranges from 0 to 45, with the lowest score representing the best mental health. All items of the DSS-4 had high factor loadings on the dimension “Dissociation” (**Table 1**) and little or no loading on the other dimensions. Consequently, the entire DSS-4 was included as a separate questionnaire. Therefore, it was not included in the final summary score. The decision was taken for several reasons: 1) the DSS-4 remained intact as a whole questionnaire in the EFAs; 2) the items of the DSS-4 were measured on a scale between 0 and 10, and thus, a high value in the items would greatly distort the summary score of the new screening instrument; and 3) the observed prevalence of dissociative symptoms was low in patients presenting at CRDs.

**Table 2:** Overview of items and scoring in the new screening instrument.

Factors	Originating scale	Item	Scale	Range	Coding
Anxiety/depression	GAD-7	Feeling nervous, anxious, or on edge	4-point	0–3	0 = not at all 1 = several days 2 = more than half the days 3 = nearly every day
		Not being able to stop or control worrying	4-point	0–3	
		Becoming easily annoyed or irritable	4-point	0–3	
		Feeling afraid, as if something awful may happen	4-point	0–3	
	PHQ-9	Feeling down, depressed, or hopeless	4-point	0–3	0 = not at all 1 = several days 2 = more than half the days 3 = nearly every day
		Thoughts that you would be better off dead or of hurting yourself	4-point	0–3	
	SCL-K-9	Emotional vulnerability	5-point	0–4	0 = not at all 1 = a little 2 = quite a bit 3 = strong 4 = very strong
Mobility/ability of daily life	SF-12	Limited in climbing several flights of stairs	3-point	0–2	0 = no, not limited at all 1 = Yes, limited a little 2 = Yes, limited a lot
		Limited in moderate activities	3-point	0–2	
	EQ-5D	Mobility	5-point	0–4	0 = no problems 1 = slight problems 2 = moderate problems 3 = severe problems 4 = being unable
		Self-care	5-point	0–4	
Energy/fatigue	PHQ-9	Trouble falling or staying asleep, or sleeping too much	4-point	0–3	0 = not at all 1 = several days 2 = more than half the days 3 = nearly every day
		Feeling tired or having little energy	4-point	0–3	
	SF-12	Having a lot of energy	6-point	0–5	0 = all of the time 1 = most of the time 2 = a good bit of the time 3 = some of the time 4 = a little of the time 5 = none of the time
Summary score				0–45	

GAD-7, 7-item Generalized Anxiety Disorder; PHQ-9, 9-item Patient Health Questionnaire; SCL-K-9, Symptom-Checklist K-9; SF-12, 12-Item Short Form Health Survey.

**Table 3** shows the internal reliability displaced as Cronbach’s α of each factor and the correlations between the individual factors. Cronbach’s α ranged from 0.61 to 0.87, suggesting acceptable to good reliability. The four factors correlated significantly positively with each other. Strong correlation was observed between the factors “anxiety/depression” and “energy/fatigue”. Moderate correlation was observed between the factors “anxiety/depression” and “dissociation”, between “mobility/ability of daily life” and “energy/fatigue”, and between “energy/fatigue” and “dissociation”. The factor correlations did not exceed 0.80, which suggests acceptable discriminant validity (28).

**Table 3:** Cronbach’s α and correlations of the extracted factors.

Factor	Cronbach’s α	Anxiety/depression	Mobility/ability of daily life	Energy/fatigue	Dissociation
Anxiety/depression	0.87	1			
Mobility/ability of daily life	0.81	0.27**	1		
Energy/fatigue	0.73	0.52**	0.36**	1	
Dissociation	0.61	0.39**	0.26**	0.31**	1

** *p*<0.01.

### 3.3 Goodness of fit of the new short screening instrument

The first CFA was based on the questionnaires on mental health filled in by both CG and IG patients at baseline (T1). The model included all items from the final set and the respective factor structure resulting from the EFA (**Supplementary Figure 1**). **Table 4** shows the parameter estimates for the items of the latent factors. The following model fit indices were calculated: χ^2^(129)=673.43 (*p*<0.001), CFI=0.94, TLI=0.92, RMSEA=0.06 (90% CI=0.05–0.06), and SRMR=0.04. The CFI was above the accepted cut-off of ≥0.90. The TLI was just below the threshold of ≥0.95. The RMSEA and the SRMR were both above the accepted cut-off of ≤0.06. All items showed positive factor loadings on their respective domains, with standardized estimates (*Beta*) ranging from 0.38 to 0.83 (**Table 4**), supporting the factor structure.

**Table 4:** Results from the first confirmatory factor analysis—parameter estimates for the items of the latent factors.

Latent factor	Item	B	S.E.	C.R.	Beta	*p*-Value
Anxiety/depression	Not being able to stop or control worrying	1.00			0.81	
Anxiety/depression	Feeling afraid, as if something awful may happen	0.83	0.03	27.66	0.73	***
Anxiety/depression	Feeling down, depressed, or hopeless	0.99	0.03	31.01	0.80	***
Anxiety/depression	Feeling nervous, anxious, or on edge	0.98	0.04	27.95	0.73	***
Anxiety/depression	Emotional vulnerability	1.02	0.04	25.48	0.68	***
Anxiety/depression	Thoughts that you would be better off dead or of hurting yourself	0.44	0.02	20.40	0.56	***
Anxiety/depression	Becoming easily annoyed or irritable	0.67	0.03	21.76	0.59	***
Mobility/ability of daily life	Mobility	1.00			0.81	
Mobility/ability of daily life	Limited in climbing several flights of stairs	0.64	0.02	27.26	0.78	***
Mobility/ability of daily life	Limited in moderate activities	0.58	0.02	26.21	0.75	
Mobility/ability of daily life	Self-care	0.59	0.03	21.58	0.62	***
Energy/fatigue	Feeling tired or having little energy	1.00			0.83	
Energy/fatigue	Having a lot of energy	1.21	0.05	23.87	0.75	***
Energy/fatigue	Trouble falling or staying asleep, or sleeping too much	0.72	0.04	18.35	0.55	***
Dissociation	Problems with hearing/sounds coming from far away	1.00			0.59	
Dissociation	Sensation that people/things/world are not real	0.76	0.06	13.80	0.58	***
Dissociation	Sensation that body/body parts are insensitive to pain	0.62	0.06	10.26	0.38	***
Dissociation	Sensation that body does not belong to you	1.62	0.11	14.35	0.65	***

B, non-standardized estimate; S.E., standard error; C.R., chi-square ratio; Beta, standardized estimate.

*** *p*<0.001.

The second CFA was based on the questionnaires on mental health filled in by IG patients at T0. The model included the same items as in the first CFA. **Table 5** shows the parameter estimates for the items of the latent factors. The following model fit indices were calculated: χ^2^(129)=304.92 (*p*<0.001), CFI=0.96, TLI=0.95, and RMSEA=0.05 (90% CI=0.04–0.05). Due to missing values, no SRMR was calculated. All three indices were above the established thresholds of ≥0.95 and ≤0.06. All items showed positive factor loadings on their respective domains, with Beta ranging from 0.50 to 0.85 (**Table 5**), supporting the factor structure.

**Table 5:** Results from the second confirmatory factor analysis—parameter estimates for the items of the latent factors.

Latent factor	Item	B	S.E.	C.R.	Beta	*p*-Value
Anxiety/depression	Not being able to stop or control worrying	1.00			0.82	
Anxiety/depression	Feeling afraid, as if something awful may happen	0.77	0.04	18.87	0.70	***
Anxiety/depression	Feeling down, depressed, or hopeless	0.99	0.04	22.66	0.81	***
Anxiety/depression	Feeling nervous, anxious, or on edge	0.99	0.05	21.06	0.76	***
Anxiety/depression	Emotional vulnerability	0.96	0.05	17.61	0.66	***
Anxiety/depression	Thoughts that you would be better off dead or of hurting yourself	0.40	0.03	13.33	0.52	***
Anxiety/depression	Becoming easily annoyed or irritable	0.65	0.04	15.14	0.58	***
Mobility/ability of daily life	Mobility	1.00			0.80	
Mobility/ability of daily life	Limited in climbing several flights of stairs	0.61	0.03	18.14	0.74	***
Mobility/ability of daily life	Limited in moderate activities	0.57	0.03	18.01	0.74	***
Mobility/ability of daily life	Self-care	0.65	0.04	16.74	0.68	***
Energy/fatigue	Feeling tired or having little energy	1.00			0.85	
Energy/fatigue	Having a lot of energy	1.24	0.07	18.03	0.77	***
Energy/fatigue	Trouble falling or staying asleep, or sleeping too much	0.69	0.06	12.73	0.53	***
Dissociation	Problems with hearing/sounds coming from far away	1.00			0.60	
Dissociation	Sensation that people/things/world are not real	0.66	0.07	10.05	0.54	***
Dissociation	Sensation that body/body parts are insensitive to pain	0.84	0.09	9.47	0.50	***
Dissociation	Sensation that body does not belong to you	1.77	0.16	11.44	0.74	***

B, non-standardized estimate; S.E., standard error; C.R., chi-square ratio; Beta, standardized estimate.

*** *p*<0.001.

### 3.4 Discriminatory power of the new short screening instrument

**Figure 4:**
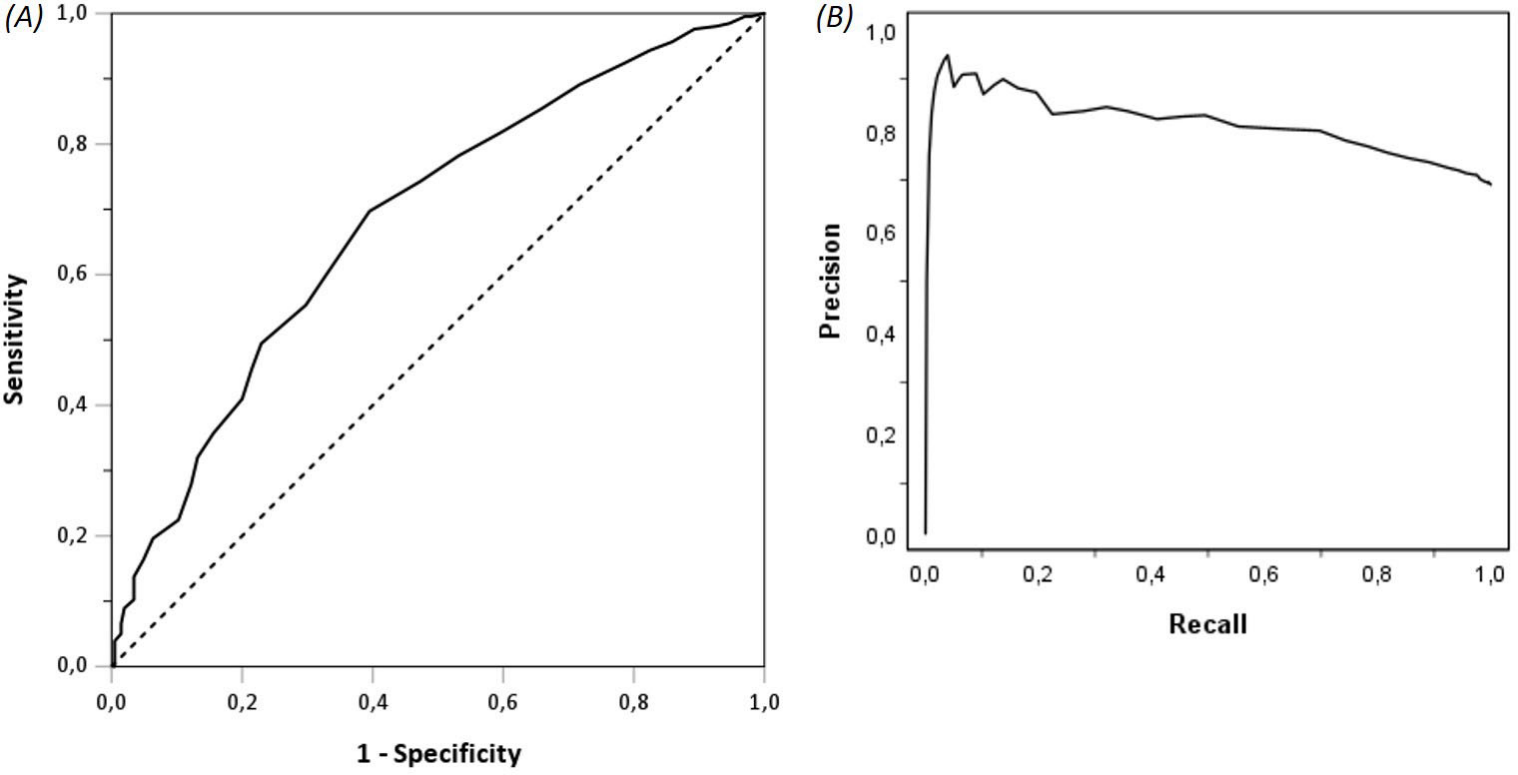
Receiver operating characteristic (ROC) curve **(A)** and precision-recall curve (PRC) **(B)** for the summary score of the new short screening instrument.

Within the follow-up process, the MHE documented at least one confirmed or suspected diagnosis of a mental disorder in 35.3% (n=459) of IG patients. The frequency of confirmed and suspected mental disorders in the IG is displayed in **Supplementary Table 4**. The predictive value of the new screening instrument for any confirmed or suspected diagnosis of a mental disorder was moderate (AUC=0.68, 95% CI=0.64–0.73), being equivalent to a Cohen’s *d* of 0.66 (medium effect) (35). The ROC curve for the summary score of the new screening instrument is presented in **Figure 4a**. The PRC is presented in **Figure 4b** with a PR-AUC of 0.81 (95% CI=0.79–0.81), being equivalent to a Cohen’s d of 1.24 (large effect) (35).

Furthermore, the predictive value of the new screening instrument was comparable or higher than for the specific standardized instruments on mental health in the IG (PHQ-9: AUC=0.69, 95% CI=0.65–0.74; GAD-7: AUC=0.67, 95% CI=0.62–0.72; SF-12 PCS: AUC=0.46, 95% CI=0.41–0.51; SF-12 MCS: AUC=0.68, 95% CI=0.63–0.72; SCL-K-9: AUC=0.69, 95% CI=0.65–0.74; EQ-5D index: AUC=0.61, 95% CI=0.57–0.66; EQ-VAS: AUC=0.61, 95% CI=0.56–0.65; DSS-4: AUC=0.61, 95% CI=0.56–0.65).

Additionally, the predictive value of the new screening instrument for depressive disorders (F30 and F33-F39) was higher than its predictive value considering any mental disorders in the IG (AUC=0.77, 95% CI=0.73–0.81), yet lower for anxiety (F40–F41: AUC=0.64, 95% CI=0.60–0.69) and somatoform/adjustment disorders (F43 and F45: AUC=0.52, 95% CI=0.46–0.59).

According to Youden’s index, the cut-off was set at 14 points with a sensitivity of 66.9% and a specificity of 61.7% (**Supplementary Table 5**, **Supplementary Figure 2**). With this cut-off point, 64.7% IG patients would be correctly identified as having a (suspected) mental disorder, and 63.9% would be correctly identified as having no such diagnosis; 35.3% had a certain or suspected mental disorder diagnosed by the MHE, yet would not be identified with the cut-off point. Eventually, 36.1% scored above the cut-off point but were not diagnosed with a mental disorder. The positive predictive value was 79.8%, and the negative predictive value was 30.9%. The chi-square test showed a statistically significant discriminatory power (*p*<0.001).

## 4 Discussion

The aim of the present study was to test the feasibility of developing and testing the psychometric properties and the predictive value of a new short screening instrument supporting MHEs in the diagnosis of mental disorders in patients presenting at CRDs. The reduction of items and the extraction of reasonable mental health dimensions using EFA from standardized questionnaires on mental health were successful. The extracted model contained 18 items, comprising the factors anxiety and depression, mobility and ability of daily life, energy and fatigue, and dissociative symptoms. The extracted model showed good fit, good reliability, and acceptable discriminant internal validity. All items except the four items of the DSS-4 were included in the new short screening instrument. The DSS-4 will remain as such in order to screen specifically for patients with potential dissociative disorders (ICD-10 F44). Of important note, the reported Cronbach’s α for the DSS-4 was below the generally accepted threshold of 0.70 (18, 19). This may be due to the low prevalence of dissociative symptoms in the study population. Therefore, it is important to assess the meaningfulness and the necessity of applying the DSS-4 in patients presenting to CRDs in further studies.

Regarding the discriminatory power of the new screening instrument, the AUC indicated a moderate discriminatory power with a medium effect for the prediction of any confirmed or suspected mental disorder. The PR-AUC indicated a good discriminatory power with a large effect. A possible explanation for this moderate effect could be the heterogeneity of the patient collective presenting at CRDs or rather the heterogeneity of the diagnosed and suspected mental disorders. A huge proportion of the diagnoses were in the group of somatoform disorders (ICD-10 F45) and adjustment disorders (ICD-10 F43). These types of conditions cannot be detected by any of the applied standardized mental health questionnaires (12, 14–19). This is supported by the observed poor discriminatory power of the new screening instrument for these disorders. However, the discriminatory power of the new screening instrument was particularly good for depressive disorders. Notably, the recurrent depressive disorder (ICD-10 F33) was the second most common mental disorder in the study population. Hence, a strength of the new screening instrument may be the identification of patients presenting at CRDs with potential depressive symptoms. Furthermore, with a cut-off set at 14 points, the majority of the patients would be correctly identified as having a (suspected) mental disorder or needing a consultation by an MHE, using the new screening instrument. Moreover, the discriminatory power of the new screening instrument for any mental disorder was comparable to or higher than that of the specific standardized instruments on mental health. This shows that even a short screening instrument can be used to identify patients in need of consultation by an MHE, reducing the burden on patients of having to complete several questionnaires.

In order to improve the new short screening instrument in this regard, screening questionnaires for somatoform and adjustment disorders such as the PHQ-15 (36), the International Adjustment Disorder Questionnaire (IAQD) (37), the SCL-90-SOM (36), or the Adjustment Disorder–New Module 20 (ADNM-20) (38) should be considered in the further development of the tool.

Another important aspect to consider in evaluating mental health and identifying patients in need of MHE consultation may be the concept of (Big Five) personality traits (39–41). Several studies have shown that personality traits, particularly conscientiousness, openness, extraversion, and neuroticism, may be associated with self-rated health, satisfaction, or resilience (39–41). Therefore, it may be important to incorporate these personality traits in the screening instrument to assess patients presenting at CRDs.

In general, a huge variety of specific questionnaires for chronic diseases on mental health or psychosomatic burden exists [e.g (42–47).,]. Furthermore, specific questionnaires exist for some RDs [e.g (48–50).,]. However, patients presenting at CRDs have symptoms of unclear origin without a clear diagnosis (4), rendering those specific questionnaires unsuitable. Additionally, in patients with suspected RDs, it is often difficult to separate somatic, psychosomatic, and mental symptoms (7, 8). This not only highlights the need for MHEs in the diagnostic course and adequate treatment of these patients but also emphasizes the need for a non-disease-specific mental health instrument covering different aspects of mental health. Therefore, the new short screening instrument may be helpful to direct the resources of the MHE to those patients in need. This approach would be consistent with the principles of resource-oriented care (51), as it would facilitate more efficient utilization of the MHE’s limited resources. The brevity of the new short screening instrument may be particularly advantageous as both time-saving and reducing the burden on patients.

### 4.1 Strengths and limitations

To the best of our knowledge, this is the first concept for a screening instrument to help detect mental disorders in patients presenting at CRDs. We have made a first attempt to assign factor names to the identified factors representing the mental health domains, taking into account the domains already specified in the standardized questionnaires. Nevertheless, the appropriateness of these names should be further investigated.

Due to the exploratory design and as the aim was to test the feasibility of developing the new screening instrument and its psychometric properties, the external validity on a different cohort with known diagnoses could not be evaluated in the present study. Furthermore, due to the COVID-19 pandemic, the factor structure could not be identified relying on the CG only. Due to the sequential study design, the majority of IG patients were recruited during the pandemic, potentially resulting in differences in the study population. Therefore, we developed the questionnaire based on data from both groups (CG and IG) and tested the psychometric properties on IG patients. By including data from both IG and CG in the analyses, the power of the results of the internal validity may be reduced. Testing the external validity on a different cohort with known diagnoses of the new screening instrument should be evaluated in further studies.

Eventually, the MHEs were not rater-blinded regarding the assessment of the mental health instruments (T0 and T1) and the documentation of the diagnoses of mental disorders. However, the latter was only documented at T2 after 12 months.

## 5 Conclusion

The design of a new short screening instrument for mental health in patients presenting at CRDs was feasible. In general, the MHEs considered the instruments on mental health to be helpful and directive for their conclusions and favored their administration prior to patients’ first CRD visit. The new short screening instrument may help MHEs to identify patients in need of a more thorough screening and adapted care, particularly in identifying patients with potential depressive disorders. However, due to the heterogeneity of mental disorders in this patient group, it was difficult to predict a specific mental disorder using the new screening instrument. Therefore, further studies should refine the new screening instrument to increase the predictive power also for other mental disorders and better support the MHEs in identifying patients who may suffer from a (co-)morbid mental disorder and may need their consultation. Moreover, further studies applying the new screening instrument together with other established questionnaires are needed to examine whether its discriminatory power for mental disorders in this patient group can be improved and to test its external validity in independent cohorts.

## Data Availability

The raw data supporting the conclusions of this article will be made available by the authors, without undue reservation.

## Author’s note

For the standardized instruments on mental health, we compiled with the copyright conditions and obtained licenses for the instruments requiring a license (SF-12, SCL-K-9) within the ZSE-DUO study. For the instruments not requiring a license (EQ-5D-5L, EQ-VAS, GAD-7, PHQ-9, DSS-4), we obtained permission to use them within the ZSE-DUO study.
