# Supplementary figures and images for: Feasibility of the development and psychometric properties of a standardized screening instrument for mental disorders in patients with suspected rare diseases: results of the ZSE-DUO study
Source: Front Psychiatry. 2025 Nov 10;16:1624474. doi: 10.3389/fpsyt.2025.1624474 (PMC12641394; doi:10.3389/fpsyt.2025.1624474)

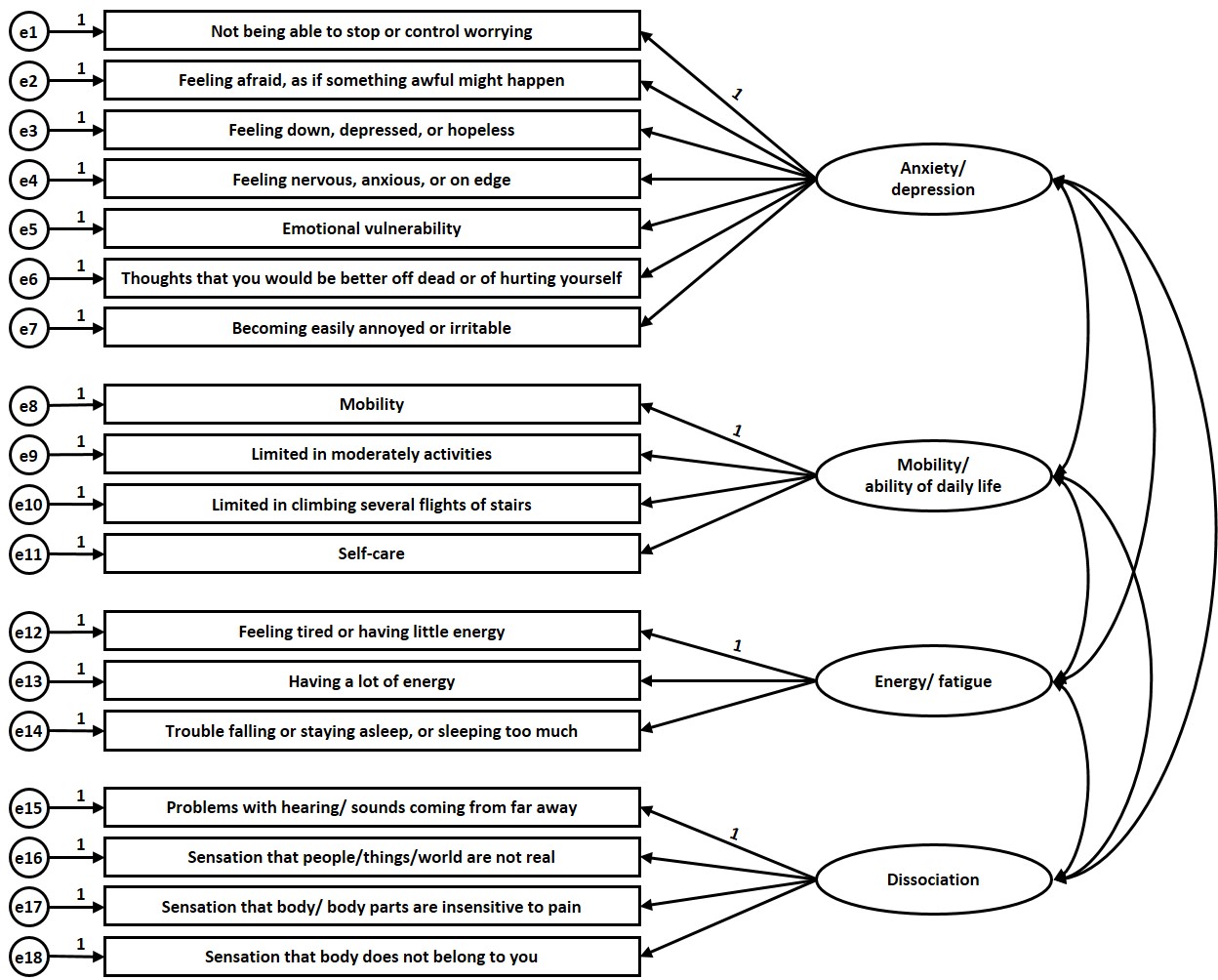

Supplement: Supplementary file 1 [file Image1.jpeg]

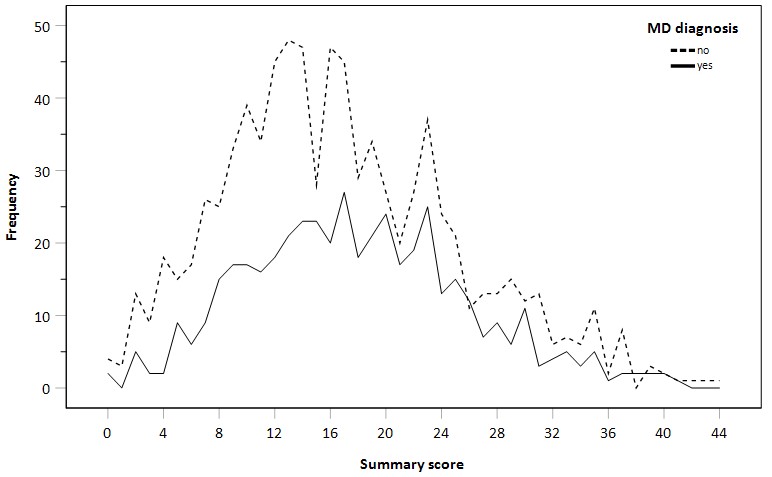

Supplement: Supplementary file 2 [file Image2.jpeg]
