# Supplementary material for: Feasibility of the development and psychometric properties of a standardized screening instrument for mental disorders in patients with suspected rare diseases: results of the ZSE-DUO study
Source: Front Psychiatry. 2025 Nov 10;16:1624474. doi: 10.3389/fpsyt.2025.1624474 (PMC12641394; doi:10.3389/fpsyt.2025.1624474)
Supplement: Supplementary file 3 [file Table1.docx]

*Supplementary Table 1. Extracted factor structure with factor loadings, rotated to the Varimax criterion (first exploratory factor analysis, all 44 items).*

| **Originating scale** | **Item** | **Factor** | | | | | | |
| --- | --- | --- | --- | --- | --- | --- | --- | --- |
|  |  | **1** | **2** | **3** | **4** | **5** | **6** |  |
| SCL-K-9 | Feeling that you worry too much | 0.802 | 0.086 | 0.144 | 0.100 | 0.067 | 0.032 |  |
| GAD-7 | Not being able to stop or control worrying | 0.792 | 0.157 | 0.130 | 0.061 | -0.029 | 0.067 |  |
| GAD-7 | Worrying to much about different things | 0.774 | 0.180 | 0.129 | 0.054 | 0.002 | 0.088 |  |
| PHQ-9 | Feeling down, depressed, or hopeless | 0.753 | 0.141 | 0.281 | 0.090 | -0.034 | -0.092 |  |
| SF-12 | Have you felt down-hearted and blue? | 0.746 | 0.177 | 0.154 | 0.070 | 0.003 | -0.206 |  |
| GAD-7 | Feeling afraid, as if something awful might happen | 0.746 | 0.059 | 0.038 | 0.178 | 0.044 | 0.030 |  |
| EQ-5D-5L | Anxiety/ depression | 0.730 | 0.247 | 0.189 | 0.012 | 0.042 | -0.094 |  |
| GAD-7 | Feeling nervous, anxious, or on edge | 0.710 | 0.165 | 0.159 | 0.092 | -0.080 | 0.164 |  |
| SCL-K-9 | Emotional vulnerability | 0.684 | 0.105 | 0.156 | 0.062 | 0.279 | 0.049 |  |
| SF-12 | Have you felt calm & peaceful? | 0.678 | 0.086 | 0.331 | 0.069 | -0.078 | 0.134 |  |
| SCL-K-9 | Uncontrollable emotional outbursts | 0.675 | 0.102 | 0.145 | 0.117 | 0.195 | -0.010 |  |
| SCL-K-9 | Feeling uptight or agitated | 0.643 | 0.044 | 0.188 | 0.104 | 0.182 | 0.210 |  |
| GAD-7 | Trouble relaxing | 0.612 | 0.117 | 0.334 | 0.072 | -0.143 | 0.296 |  |
| PHQ-9 | Feeling bad about yourself — or that you are a failure or have let yourself or your family down | 0.595 | 0.026 | 0.149 | 0.116 | 0.365 | -0.056 |  |
| PHQ-9 | Little interest or pleasure in doing things | 0.591 | 0.146 | 0.353 | 0.109 | -0.039 | -0.058 |  |
| SCL-K-9 | Feelings of loneliness even in company | 0.571 | 0.026 | 0.156 | 0.146 | 0.415 | -0.224 |  |
| GAD-7 | Becoming easily annoyed or irritable | 0.567 | 0.080 | 0.195 | 0.064 | 0.075 | 0.284 |  |
| PHQ-9 | Thoughts that you would be better off dead or of hurting yourself in some way | 0.549 | 0.124 | 0.060 | 0.178 | 0.212 | -0.334 |  |
| SCL-K-9 | Feeling nervous when left to yourself | 0.546 | 0.139 | 0.029 | 0.179 | 0.322 | 0.030 |  |
| SCL-K-9 | Feeling it difficult to start something | 0.532 | 0.190 | 0.408 | 0.136 | 0.149 | -0.004 |  |
| Life happiness | Life happiness | 0.435 | 0.327 | 0.295 | 0.008 | 0.028 | -0.341 |  |
| EQ-5D-5L | Mobility | 0.104 | 0.827 | -0.039 | 0.040 | 0.143 | 0.058 |  |
| SF-12 | Limited in moderately activities | 0.052 | 0.763 | 0.170 | 0.060 | 0.097 | -0.030 |  |
| EQ-5D-5L | Usual activities | 0.165 | 0.743 | 0.247 | 0.038 | 0.006 | -0.083 |  |
| SF-12 | Limited in climbing several flights of stairs | -0.016 | 0.739 | 0.101 | 0.047 | 0.200 | 0.071 |  |
| EQ-5D-5L | Self-care | 0.138 | 0.668 | -0.094 | 0.070 | 0.240 | 0.080 | |
| SF-12 | How much did pain interfere with your normal work? | 0.181 | 0.632 | 0.277 | 0.123 | -0.243 | 0.119 | |
| SF-12 | In general, would you say your health is…? | 0.220 | 0.603 | 0.380 | 0.038 | -0.194 | -0.117 | |
| EQ-5D-5L | Pain/discomfort | 0.193 | 0.592 | 0.198 | 0.124 | -0.339 | 0.058 | |
| EQ-VAS | EQ Visual Analogue Scale | 0.265 | 0.584 | 0.292 | 0.075 | -0.182 | -0.138 | |
| SCL-K-9 | Feeling of heaviness in your arms or legs | 0.150 | 0.450 | 0.211 | 0.306 | 0.046 | 0.300 | |
| PHQ-9 | Feeling tired or having little energy | 0.228 | 0.228 | 0.753 | 0.077 | 0.037 | 0.069 | |
| SF-12 | Did you have a lot of energy? | 0.251 | 0.295 | 0.701 | 0.048 | -0.005 | -0.129 | |
| PHQ-9 | Trouble concentrating on things, such as reading the newspaper or watching television | 0.320 | 0.119 | 0.573 | 0.187 | 0.204 | 0.046 | |
| PHQ-9 | Trouble falling or staying asleep, or sleeping too much | 0.261 | 0.164 | 0.552 | 0.039 | -0.015 | 0.206 | |
| SF-12 | How much of the time has your physical health or emotional problems interfered with your social activities (like visiting friends, relatives, etc.)? | 0.303 | 0.361 | 0.455 | 0.049 | 0.091 | -0.177 | |
| PHQ-9 | Poor appetite or overeating | 0.287 | 0.089 | 0.394 | 0.210 | 0.133 | 0.168 | |
| DSS-4 | Problems with hearing/ sounds coming from far away | 0.100 | 0.062 | 0.135 | 0.730 | 0.103 | -0.008 | |
| DSS-4 | Sensation that people/things/world are not real | 0.209 | 0.007 | 0.074 | 0.699 | 0.041 | -0.234 | |
| DSS-4 | Sensation that body does not belong to you | 0.270 | 0.220 | 0.137 | 0.583 | 0.034 | 0.077 | |
| DSS-4 | Sensation that body/ body parts are insensitive to pain | 0.035 | 0.091 | -0.004 | 0.578 | -0.010 | 0.163 | |
| SCL-K-9 | Feeling observed and talked about | 0.423 | 0.052 | 0.080 | 0.088 | 0.576 | 0.025 | |
| PHQ-9 | Moving or speaking so slowly that other people could have noticed? Or the opposite — being so fidgety or restless that you have been moving around a lot more than usual | 0.158 | 0.371 | 0.218 | 0.127 | 0.437 | 0.301 | |
| GAD-7 | Being so restless that it is hard to sit still | 0.455 | 0.133 | 0.188 | 0.118 | 0.117 | 0.502 | |
