# Supplementary material for: Feasibility of the development and psychometric properties of a standardized screening instrument for mental disorders in patients with suspected rare diseases: results of the ZSE-DUO study
Source: Front Psychiatry. 2025 Nov 10;16:1624474. doi: 10.3389/fpsyt.2025.1624474 (PMC12641394; doi:10.3389/fpsyt.2025.1624474)
Supplement: Supplementary file 4 [file Table2.docx]

*Supplementary Table 2. Extracted factor structure with factors loadings, rotated to the Varimax criterion (second exploratory factor analysis, reduced items/ n=28).*

| **Originating scale** | **Item** | **Factor** | | | |
| --- | --- | --- | --- | --- | --- |
|  |  | **1** | **2** | **3** | **4** |
| SCL-K-9 | Feeling that you worry too much | 0.814 | 0.057 | 0.145 | 0.107 |
| GAD-7 | Not being able to stop or control worrying | 0.798 | 0.136 | 0.143 | 0.048 |
| GAD-7 | Worrying too much about different things | 0.787 | 0.153 | 0.153 | 0.044 |
| PHQ-9 | Feeling down, depressed, or hopeless | 0.754 | 0.112 | 0.273 | 0.081 |
| GAD-7 | Feeling afraid, as if something awful might happen | 0.747 | 0.046 | 0.048 | 0.172 |
| EQ-5D-5L | Anxiety/ depression | 0.744 | 0.229 | 0.182 | 0.006 |
| SF-12 | Have you felt down-hearted and blue? | 0.743 | 0.152 | 0.146 | 0.063 |
| SCL-K-9 | Emotional vulnerability | 0.734 | 0.089 | 0.115 | 0.094 |
| SCL-K-9 | Uncontrollable emotional outbursts | 0.710 | 0.089 | 0.096 | 0.147 |
| GAD-7 | Feeling nervous, anxious, or on edge | 0.702 | 0.144 | 0.187 | 0.083 |
| SCL-K-9 | Feeling uptight or agitated | 0.677 | 0.023 | 0.148 | 0.124 |
| GAD-7 | Becoming easily annoyed or irritable | 0.588 | 0.054 | 0.201 | 0.083 |
| PHQ-9 | Thoughts that you would be better off dead or of hurting  yourself in some way | 0.586 | 0.134 | -0.026 | 0.191 |
| EQ-5D-5L | Mobility | 0.125 | 0.844 | 0.008 | 0.053 |
| SF-12 | Limited in moderately activities | 0.074 | 0.763 | 0.208 | 0.087 |
| SF-12 | Limited in climbing several flights of stairs | 0.030 | 0.749 | 0.122 | 0.083 |
| EQ-5D-5L | Usual activities | 0.178 | 0.733 | 0.286 | 0.049 |
| EQ-5D-5L | Self-care | 0.176 | 0.718 | -0.144 | 0.099 |
| SF-12 | How much did pain interfere with your normal work? | 0.134 | 0.560 | 0.476 | 0.076 |
| EQ-VAS | EQ Visual Analogue Scale | 0.241 | 0.529 | 0.383 | 0.061 |
| EQ-5D-5L | Pain/ discomfort | 0.118 | 0.517 | 0.456 | 0.054 |
| PHQ-9 | Feeling tired or having little energy | 0.278 | 0.159 | 0.760 | 0.112 |
| SF-12 | Did you have a lot of energy? | 0.286 | 0.246 | 0.655 | 0.091 |
| PHQ-9 | Trouble falling or staying asleep, or sleeping too much | 0.264 | 0.100 | 0.638 | 0.072 |
| DSS-4 | Problems with hearing/ sounds coming from far away | 0.125 | 0.062 | 0.091 | 0.748 |
| DSS-4 | Sensation that people/things/world are not real | 0.215 | 0.005 | 0.036 | 0.698 |
| DSS-4 | Sensation that body/ body parts are insensitive to pain | 0.015 | 0.090 | 0.020 | 0.594 |
| DSS-4 | Sensation that body does not belong to you | 0.276 | 0.193 | 0.153 | 0.592 |
