# Supplementary material for: Feasibility of the development and psychometric properties of a standardized screening instrument for mental disorders in patients with suspected rare diseases: results of the ZSE-DUO study
Source: Front Psychiatry. 2025 Nov 10;16:1624474. doi: 10.3389/fpsyt.2025.1624474 (PMC12641394; doi:10.3389/fpsyt.2025.1624474)
Supplement: Supplementary file 5 [file Table3.docx]

*Supplementary Table 3. Extracted factor structure with factor loadings, rotated to the Varimax criterion (third exploratory factor analysis, reduced items/ n=25).*

| **Originating scale** | **Item** | **Factor** | | | |
| --- | --- | --- | --- | --- | --- |
|  |  | **1** | **2** | **3** | **4** |
| SCL-K-9 | Feeling that you worry too much | 0.816 | 0.042 | 0.137 | 0.113 |
| GAD-7 | Not being able to stop or control worrying | 0.802 | 0.122 | 0.133 | 0.056 |
| GAD-7 | Worrying too much about different things | 0.790 | 0.140 | 0.150 | 0.051 |
| PHQ-9 | Feeling down, depressed, or hopeless | 0.757 | 0.100 | 0.263 | 0.088 |
| GAD-7 | Feeling afraid, as if something awful might happen | 0.750 | 0.028 | 0.032 | 0.180 |
| EQ-5D-5L | Anxiety/ depression | 0.747 | 0.221 | 0.184 | 0.013 |
| SF-12 | Have you felt down-hearted and blue? | 0.747 | 0.137 | 0.132 | 0.072 |
| SCL-K-9 | Emotional vulnerability | 0.731 | 0.082 | 0.129 | 0.097 |
| SCL-K-9 | Uncontrollable emotional outbursts | 0.713 | 0.074 | 0.088 | 0.153 |
| GAD-7 | Feeling nervous, anxious, or on edge | 0.705 | 0.131 | 0.181 | 0.091 |
| SCL-K-9 | Feeling uptight or agitated | 0.674 | 0.013 | 0.155 | 0.127 |
| GAD-7 | Becoming easily annoyed or irritable | 0.587 | 0.048 | 0.209 | 0.085 |
| PHQ-9 | Thoughts that you would be better off dead or of hurting  yourself in some way | 0.584 | 0.123 | -0.022 | 0.198 |
| EQ-5D-5L | Mobility | 0.131 | 0.850 | 0.032 | 0.056 |
| SF-12 | Limited in moderately activities | 0.082 | 0.770 | 0.226 | 0.087 |
| SF-12 | Limited in climbing several flights of stairs | 0.025 | 0.769 | 0.184 | 0.078 |
| EQ-5D-5L | Self-care | 0.172 | 0.735 | -0.097 | 0.096 |
| EQ-5D-5L | Usual activities | 0.193 | 0.728 | 0.280 | 0.057 |
| PHQ-9 | Feeling tired or having little energy | 0.267 | 0.171 | 0.815 | 0.109 |
| SF-12 | Did you have a lot of energy? | 0.274 | 0.258 | 0.712 | 0.089 |
| PHQ-9 | Trouble falling or staying asleep, or sleeping too much | 0.265 | 0.100 | 0.650 | 0.074 |
| DSS-4 | Problems with hearing/ sounds coming from far away | 0.118 | 0.056 | 0.099 | 0.748 |
| DSS-4 | Sensation that people/things/world are not real | 0.210 | -0.005 | 0.033 | 0.701 |
| DSS-4 | Sensation that body does not belong to you | 0.277 | 0.186 | 0.144 | 0.596 |
| DSS-4 | Sensation that body/ body parts are insensitive to pain | 0.015 | 0.088 | 0.006 | 0.595 |
