# Supplementary material for: Feasibility of the development and psychometric properties of a standardized screening instrument for mental disorders in patients with suspected rare diseases: results of the ZSE-DUO study
Source: Front Psychiatry. 2025 Nov 10;16:1624474. doi: 10.3389/fpsyt.2025.1624474 (PMC12641394; doi:10.3389/fpsyt.2025.1624474)
Supplement: Supplementary file 6 [file Table4.docx]

*Supplementary Table 4. Frequency of suspected and diagnosed mental disorders in the intervention group of the ZSE-DUO study (n=664).*

| **ICD-10 code** | **Description** | **Frequency**  **(n, %)*** |
| --- | --- | --- |
| F45 | Somatoform disorders | 252 (38.0) |
| F33 | Recurrent depressive disorder | 101 (15.2) |
| F43 | Reaction to severe stress, and adjustment disorders | 95 (14.3) |
| F40 | Phobic anxiety disorders | 68 (10.2) |
| F32 | Depressive episode | 55 (8.3) |
| F54 | Psychological and behavioural factors associated with disorders or diseases classified elsewhere | 48 (7.2) |
| F41 | Other anxiety disorders | 47 (7.1) |
| F17 | Mental and behavioural disorders due to use of tobacco | 23 (3.5) |
| F44 | Dissociative [conversion] disorders | 20 (3.0) |
| F51 | Nonorganic sleep disorders | 17 (2.6) |
| F34 | Persistent mood [affective] disorders | 16 (2.4) |
| F50 | Eating disorders | 16 (2.4) |
| F42 | Obsessive-compulsive disorder | 14 (2.1) |
| F48 | Other neurotic disorders | 14 (2.1) |
| F10 | Mental and behavioural disorders due to use of alcohol | 12 (1.8) |
| F60 | Specific personality disorders | 12 (1.8) |
| F61 | Mixed and other personality disorders | 8 (1.2) |
| F31 | Bipolar affective disorder | 7 (1.1) |
| F07 | Personality and behavioural disorders due to brain disease, damage and dysfunction | 5 (0.8) |
| F13 | Mental and behavioural disorders due to use of sedatives or hypnotics | 5 (0.8) |
| F52 | Sexual dysfunction, not caused by organic disorder or disease | 5 (0.8) |
| F70 | Mild mental retardation | 5 (0.8) |
| F06 | Other mental disorders due to brain damage and dysfunction and to physical disease | 4 (0.6) |
| F12 | Mental and behavioural disorders due to use of cannabinoids | 4 (0.6) |
| F68 | Other disorders of adult personality and behaviour | 4 (0.6) |
| F20 | Schizophrenia | 3 (0.5) |
| F90 | Hyperkinetic disorders | 3 (0.5) |
| F98 | Other behavioural and emotional disorders with onset usually occurring in childhood and adolescence | 3 (0.5) |
| F02 | Dementia in other diseases classified elsewhere | 2 (0.3) |
| F19 | Mental and behavioural disorders due to multiple drug use and use of other psychoactive substances | 2 (0.3) |
| F21 | Schizotypal disorder | 2 (0.3) |
| F22 | Persistent delusional disorders | 2 (0.3) |
| F63 | Habit and impulse disorders | 2 (0.3) |
| F83 | Mixed specific developmental disorders | 2 (0.3) |
| F84 | Pervasive developmental disorders | 2 (0.3) |
| F03 | Unspecified dementia | 1 (0.2) |
| F05 | Delirium, not induced by alcohol and other psychoactive substances | 1 (0.2) |
| F11 | Mental and behavioural disorders due to use of opioids | 1 (0.2) |
| F23 | Acute and transient psychotic disorders | 1 (0.2) |
| F30 | Manic episode | 1 (0.2) |
| F55 | Abuse of non-dependence-producing substances | 1 (0.2) |
| F72 | Severe mental retardation | 1 (0.2) |
| F81 | Specific developmental disorders of scholastic skills | 1 (0.2) |
| ** As several mental disorders could be diagnosed per patient, the number does not add up to 664.* | | |
