# Supplementary material for: Feasibility of the development and psychometric properties of a standardized screening instrument for mental disorders in patients with suspected rare diseases: results of the ZSE-DUO study
Source: Front Psychiatry. 2025 Nov 10;16:1624474. doi: 10.3389/fpsyt.2025.1624474 (PMC12641394; doi:10.3389/fpsyt.2025.1624474)
Supplement: Supplementary file 7 [file Table5.docx]

*Supplementary Table 5. Tested cut-off values, sensitivity, specificity and calculated Youden index.*

| **Cut-off for summary score** | **Sensitivity** | **1-Specificity** | **Specificity** | **Specificity-1** | **Youden index** |
| --- | --- | --- | --- | --- | --- |
| -1.000 | 1.000 | 1.000 | 0.000 | -1.000 | 0.000 |
| 0.500 | 0.995 | 0.979 | 0.021 | -0.979 | 0.016 |
| 1.500 | 0.995 | 0.969 | 0.031 | -0.969 | 0.027 |
| 2.500 | 0.984 | 0.948 | 0.052 | -0.948 | 0.036 |
| 3.500 | 0.979 | 0.932 | 0.068 | -0.932 | 0.047 |
| 4.500 | 0.974 | 0.896 | 0.104 | -0.896 | 0.078 |
| 5.500 | 0.953 | 0.859 | 0.141 | -0.859 | 0.094 |
| 6.500 | 0.941 | 0.833 | 0.167 | -0.833 | 0.108 |
| 7.500 | 0.920 | 0.792 | 0.208 | -0.792 | 0.129 |
| 8.500 | 0.890 | 0.724 | 0.276 | -0.724 | 0.166 |
| 9.500 | 0.850 | 0.672 | 0.328 | -0.672 | 0.178 |
| 10.500 | 0.815 | 0.609 | 0.391 | -0.609 | 0.205 |
| 11.500 | 0.779 | 0.542 | 0.458 | -0.542 | 0.238 |
| 12.500 | 0.742 | 0.479 | 0.521 | -0.479 | 0.263 |
| 13.500 | 0.695 | 0.401 | 0.599 | -0.401 | 0.294 |
| 14.500 | 0.643 | 0.365 | 0.635 | -0.365 | 0.279 |
| 15.500 | 0.594 | 0.328 | 0.672 | -0.328 | 0.266 |
| 16.500 | 0.547 | 0.302 | 0.698 | -0.302 | 0.245 |
| 17.500 | 0.493 | 0.229 | 0.771 | -0.229 | 0.264 |
| 18.500 | 0.455 | 0.219 | 0.781 | -0.219 | 0.237 |
| 19.500 | 0.408 | 0.203 | 0.797 | -0.203 | 0.205 |
| 20.500 | 0.359 | 0.161 | 0.839 | -0.161 | 0.198 |
| 21.500 | 0.329 | 0.141 | 0.859 | -0.141 | 0.188 |
| 22.500 | 0.284 | 0.130 | 0.870 | -0.130 | 0.154 |
| 23.500 | 0.228 | 0.109 | 0.891 | -0.109 | 0.118 |
| 24.500 | 0.197 | 0.068 | 0.932 | -0.068 | 0.129 |
| 25.500 | 0.162 | 0.052 | 0.948 | -0.052 | 0.110 |
| 26.500 | 0.141 | 0.036 | 0.964 | -0.036 | 0.104 |
| 27.500 | 0.127 | 0.036 | 0.964 | -0.036 | 0.090 |
| 28.500 | 0.106 | 0.036 | 0.964 | -0.036 | 0.069 |
| 29.500 | 0.092 | 0.021 | 0.979 | -0.021 | 0.071 |
| 30.500 | 0.068 | 0.016 | 0.984 | -0.016 | 0.052 |
| 31.500 | 0.061 | 0.016 | 0.984 | -0.016 | 0.045 |
| 32.500 | 0.052 | 0.016 | 0.984 | -0.016 | 0.036 |
| 33.500 | 0.040 | 0.005 | 0.995 | -0.005 | 0.035 |
| 34.500 | 0.033 | 0.005 | 0.995 | -0.005 | 0.028 |
| 35.500 | 0.023 | 0.005 | 0.995 | -0.005 | 0.018 |
| 36.500 | 0.021 | 0.005 | 0.995 | -0.005 | 0.016 |
| 37.500 | 0.016 | 0.005 | 0.995 | -0.005 | 0.011 |
| 38.500 | 0.012 | 0.005 | 0.995 | -0.005 | 0.007 |
| 39.500 | 0.007 | 0.005 | 0.995 | -0.005 | 0.002 |
| 40.500 | 0.002 | 0.005 | 0.995 | -0.005 | -0.003 |
| 41.500 | 0.000 | 0.005 | 0.995 | -0.005 | -0.005 |
| 43.000 | 0.000 | 0.000 | 1.000 | 0.000 | 0.000 |
| *The highest Youden indices are highlighted in grey.* | | | | | |
