# Supplementary material for: Feasibility of the development and psychometric properties of a standardized screening instrument for mental disorders in patients with suspected rare diseases: results of the ZSE-DUO study
Source: Front Psychiatry. 2025 Nov 10;16:1624474. doi: 10.3389/fpsyt.2025.1624474 (PMC12641394; doi:10.3389/fpsyt.2025.1624474)
Supplement: Supplementary file 8 [file Table6.docx]

*Supplementary Table 1. Members of the ZSE-DUO working group (in alphabetical order).*

Frederica Akkaya, Ulm University Medical Center; Christine Babka, Hannover Medical School; Lisa Bannert, University Hospital Regensburg; Lavinia Bârlescu, Ulm University Medical Center; Anja Bärsch-Michelmann, IKK gesund plus; Leoni Böhm, RWTH Aachen University Hospital; Folke Brinkmann, University Children’s Hospital, Ruhr University Bochum; Vanessa Britz, University Hospital Frankfurt; Monika Bullinger, University Medical Center Hamburg-Eppendorf; Holger Cario, Ulm University Medical Center; Moritz de Greck, University Hospital Frankfurt; Klaus-Michael Debatin, Ulm University Medical Center; Katrin Dillmann-Jehn, University of Tübingen; Jutta Eymann, University of Tübingen; Vega Gödecke, Hannover Medical School; Holm Graessner, University of Tübingen; Corinna Grasemann, University Children’s Hospital, Ruhr University Bochum; Harald Gündel, Ulm University Medical Center; Astrid Haas, Hannover Medical School; Lea Haisch, University Hospital Münster; Isabel Heinrich, University Medical Centre Mainz; Melissa Held, University Hospital Würzburg; Julia Hennermann, University Medical Centre Mainz; Stephan Herpertz, Ruhr University Bochum; Julian Hett, Hannover Medical School; Bettina Hilbig, Ulm University Medical Center; Laura Holthöfer, University Medical Centre Mainz; Florian Junne, University Hospital Magdeburg; Jan Kassubek, Ulm University Medical Center: Kevon-Thomas Koschitzki, University Hospital Regensburg; Heike Krassort, University Hospital Münster; Birgit Kropff, University Hospital Münster; Julia Kuhn, Ulm University Medical Center; Philipp Latzko, University Hospital Münster; Thomas Lücke, Ruhr University Bochum; Albert Ludolph, Ulm University Medical Center; Tim Maisch, University Hospital Regensburg; Eva Metzger, University Hospital Würzburg; Thorsten Meyer, Techniker Krankenkasse; Isabell Meyer dos Santos, University Hospital Frankfurt; Klaus Mohnike, University Hospital Magdeburg; Martin Mücke, RWTH Aachen University Hospital; Susanne Müller, Ulm University Medical Center; Christine Mundlos, Allianz Chronischer Seltener Erkrankungen (ACHSE) e.V., c/o DRK Kliniken Berlin Mitte; Thomas Musacchio, University Hospital Würzburg; Anja Singer, IKK gesund plus; Merial Nöhre, Hannover Medical School; Andrea Petermann-Meyer, RWTH Aachen University Hospital; Christina Pfeifer-Duck, University Medical Centre Mainz; Annika Philipps, University of Tübingen; Lea Sophie Piduhn, University Hospital Münster; Julia Quitmann, University Medical Center Hamburg-Eppendorf; Olaf Riess, University of Tübingen; Frank Rutsch, Münster University Hospital; Kristina Schaubert, Hannover Medical School; Christopher Schippers, RWTH Aachen University Hospital; Annika Schmidt, RWTH Aachen University Hospital; Ludger Schoels, University of Tübingen; Katharina Schubert, University Hospital Magdeburg; Martina Schwalba, Hannover Medical School; Susann Schweiger, University Medical Centre Mainz; Udo Selig, University Würzburg; Alexandra Sroka, University Hospital Magdeburg; Toni Steinbüchel, LWL University Hospital Bochum; Andreas Stengel, University of Tübingen; Sebastian Stösser, Ulm University Medical Center; Steffi Suchant, Techniker Krankenkasse; Daniela Volk, RWTH Aachen University Hospital; Christoph Vollmuth, University Hospital Würzburg; Solange Volnov, RWTH Aachen University Hospital; Thomas O. F. Wagner, University Hospital Frankfurt; Sabrina Walter, University Hospital Frankfurt; Bodo Warrings, University Hospital Würzburg; Kamil Zajt, RWTH Aachen University Hospital; Lena Zeltner, University Hospital Tübingen; Karola Zenker, University Hospital Magdeburg; Daving Zhang, RWTH Aachen University Hospital
